# Patients prefer human psychiatrists over chatbots: a letter to the editor

**DOI:** 10.3325/cmj.2025.66.179

**Published:** 2025-04

**Authors:** Hinpetch Daungsupawong, Viroj Wiwanitkkit

**Affiliations:** 1 Private academic consultant, Phonhong, Laos; 2 Chandigarh University, Chandigarh, India

To the Editor:

We read with interest the article “Patients Prefer Human Psychiatrists over Chatbots: A Cross-Sectional Study” published in the February issue of the *Croatian Medical Journal* (1). This study rated psychiatric patients' satisfaction with the accuracy and utility of information supplied by psychiatrists, pharmacists, ChatGPT, and a Croatian chatbot. The purpose of this cross-sectional study was to determine the relationship between the types of information presented and patient satisfaction. We have to point out several limitations of the study. The survey questions were not clearly defined, which could have led to conflicting responses from respondents. As the study was limited to people receiving therapy from institutions in two countries, it may not represent the general population in all locations. To assess the differences in satisfaction scores between the groups, proper statistical tests should have been performed. However, the report does not specify the statistical procedures used to determine the disparities. Furthermore, the association between satisfaction and characteristics such as age, gender, or disease severity was not investigated, which may have influenced the interpretation of the findings.

A broader conversation should be started on how to measure satisfaction at a deeper level than merely by providing an average score, such as by comparing the perceived information quality between patients who used a chatbot and those who preferred in-person consultations. Furthermore, issues about the emotional support and contentment with information offered by chatbots vs human consultations may be raised.

Longitudinal studies should also be performed to investigate how patient satisfaction varies over time while obtaining information from multiple sources on a constant basis. Future research could look into the role of chatbots in long-term therapy processes or their effect in the treatment of patients with severe psychiatric issues. Furthermore, it should investigate how to enhance chatbots to better answer complicated inquiries about emotional or mental circumstances, and how AI models could be better customized to the mental health setting.
